# Prenatal MRI features of fetal complete agenesis of the corpus callosum associated with unilateral hemispheric cortical malformation: a retrospective study

**DOI:** 10.3389/fradi.2026.1802335

**Published:** 2026-05-01

**Authors:** Huihui Lin, Xiaoyu Wang, Chang Wang, Gengwu Li, Xu Li

**Affiliations:** Department of Radiology, Anhui Provincial Children’s Hospital, Hefei, China

**Keywords:** agenesis of the corpus callosum, fetus, magnetic resonance imaging, ultrasound, unilateral hemispheric cortical malformation

## Abstract

**Introduction:**

To explore the prenatal MRI features of fetal complete agenesis of the corpus callosum (cACC) associated with unilateral hemispheric cortical malformation.

**Methods:**

A retrospective analysis was conducted on 101 cases diagnosed with cACC via prenatal MRI. Among them, 16 cases were found to be associated with unilateral hemispheric cortical malformation. The imaging characteristics of this specific malformation were analyzed, and its associations with fetal gender and gestational age were investigated.

**Results:**

In this group of 16 cases of cACC with unilateral cortical malformation, 13 fetuses were male and 3 were female. The gestational age at diagnosis ranged from 23 weeks to 27 weeks and 2 days. The malformation was located in the left hemisphere in 7 cases and in the right hemisphere in 9 cases. Type I (C1 type: extensive cortical twisting/folding) was observed in 11 cases, all of which exhibited the “rake sign” and “garland sign”. The “rake sign” manifests as abnormally folded dysplastic cortex resembling a rake on axial or coronal views, while the “garland sign” appears as abnormally folded dysplastic cortex resembling a garland on sagittal views. Type II (C4 type: transcortical cleft formation) was observed in 1 case, and Type III (C5 type: focal cortical indentation or serrated changes) was observed in 4 cases. The unilateral cortical malformation involved the frontal lobe in 16 cases, the parietal lobe in 10 cases, and the occipital lobe in 8 cases, with no involvement of the temporal lobe.

**Discussion:**

Fetal agenesis of the corpus callosum has a relatively high probability of being associated with unilateral cortical malformation. This malformation occurs more frequently in males, is mostly detected during the mid-trimester, and most commonly involves the frontal lobe. The “rake sign” and “garland sign” are its characteristic imaging features. Prenatal definitive diagnosis aids in the perinatal management of this malformation. Prenatal MRI is a crucial supplementary examination for fetal cACC diagnosed by prenatal ultrasound, as it can accurately detect associated unilateral hemispheric cortical malformations that are easily missed by ultrasound, and thus is essential for optimizing perinatal management and parental genetic counseling.

## 1 Introduction

Agenesis of the corpus callosum (ACC) is a rare congenital malformation of cerebral structural development, which is classified into complete or partial agenesis. It is often accompanied by other abnormalities, such as midline anomalies (e.g., interhemispheric cysts, lipomas, and absence of the septum pellucidum), posterior fossa anomalies, cortical malformations, and abnormal neuronal migration (1). With an incidence of approximately 1 in 4,000 live births, ACC exhibits marked clinical heterogeneity in its manifestations, ranging from asymptomatic presentations in isolated cases to severe neurodevelopmental impairments involving epilepsy, cognitive delay, and behavioral disorders in those with combined structural or genetic abnormalities (2). Complete ACC (cACC), in particular, has a well-documented strong association with cortical malformations, and the prognosis of fetuses with ACC is predominantly determined by these accompanying anomalies rather than the ACC itself (3, 4). As highlighted in a recent narrative review on the multidisciplinary management of ACC, accurate prenatal characterization of associated malformations is the cornerstone of effective parental counseling, prognostic assessment, and perinatal management planning—underscoring the clinical urgency of clarifying the imaging features of cACC combined with rare cortical malformation subtypes (5).

Prenatal ultrasound (US) is the primary screening method for fetal ACC. However, it is affected by many factors and prone to missed diagnosis, with a low detection rate for cortical abnormalities. Prenatal magnetic resonance imaging (MRI) is recognized as the gold standard for diagnosing fetal cortical malformations, offering superior spatial resolution to ultrasound for visualizing the fine structural development of the fetal cerebral cortex, including sulcal and gyral formation (6). This advanced imaging capability enables the precise characterization of cortical malformations associated with ACC, which is essential for the multidisciplinary team to assess fetal prognosis, guide parental decision-making, and optimize perinatal management strategies (2).

Despite this, the specific prenatal MRI features of cACC combined with unilateral hemispheric cortical malformation—a distinct and understudied subtype of associated anomalies—remain poorly elucidated in the existing literature, with limited data on its epidemiological correlates, characteristic imaging signs, and clinical outcomes.

Previous studies have emphasized the need for detailed cortical evaluation in all fetuses diagnosed with cACC (7), and a novel classification system for fetal cortical formation abnormalities has identified specific unilateral cortical malformation subtypes that are closely linked to cACC (8). To address the research gap in the imaging characterization of this complex malformation, we conducted a retrospective analysis of fetal cases with cACC and concurrent unilateral hemispheric cortical malformation diagnosed by prenatal MRI at our center. This study aims to systematically describe the prenatal MRI features, including the characteristic imaging signs, anatomical distribution, and subtype classification of the associated unilateral cortical malformations, and to explore their correlations with fetal gender and gestational age at diagnosis. By refining the prenatal MRI diagnostic criteria for this condition, we seek to improve the accuracy of prenatal diagnosis, provide more comprehensive prognostic information for multidisciplinary counseling, and ultimately inform the perinatal management of fetuses with this complex CNS malformation.

## 2 Materials and methods

### 2.1 General information

A retrospective analysis was conducted on 101 fetuses diagnosed with cACC by prenatal MRI in our hospital from August 2017 to December 2025, among which 16 fetuses were complicated with unilateral hemispheric cortical malformation, all of which were singleton pregnancies. The maternal age ranged from 26 to 45 years. The gestational age ranged from 23 weeks to 27 weeks and 2 days. Inclusion criteria: unilateral hemispheric cortical malformation (16 Cases); Exclusion criteria: ① cACC without additional cortical abnormalities (63 cases); ② bilateral cortical malformation (4 cases); ③ With specific cortical malformations: polymicrogyria, hemimegalencephaly, lissencephaly (3 cases); ④ unsatisfactory MRI image quality (15 cases).

### 2.2 Examination equipment and methods

All studies were performed using a Philips Achieva 1.5T MRI scanner. Pregnant women were instructed to lie supine with quiet breathing, without the use of sedatives. They entered the scanner head-first or feet-first, using an abdominal 16-channel coil. The scanning sequences included spin-echo T1WI, single-shot fast spin-echo (ssFSE), balanced turbo field echo (BTFE), and diffusion-weighted imaging (DWI) (b = 0, 800 s/mm^2^), with axial, coronal, and sagittal cranial scans in three orthogonal planes. Scanning parameters: For the ssFSE sequence, TR = 25,000–35,000 ms, TE = 1683 ms, slice thickness = 2.0–4.0 mm, slice gap = −3–0 mm; for the BTFE sequence, TR = 3.83 ms, TE = 1.91 ms; field of view (FOV) = 350 mm × 350 mm, matrix = 238 × 238, slice thickness = 5.0–6.0 mm, slice gap = −3–0 mm. Routine ssFSE scanning of the fetal thorax and abdomen in three orthogonal planes was performed. For fetuses with poor image quality, repeated scanning was conducted until the images were clear enough to meet the diagnostic requirements. The diagnostic criteria for complete agenesis of the corpus callosum were based on imaging findings: Complete absence of all parts of the corpus callosum, including the rostrum, genu, body, and splenium, without any visible fibrous connection between the bilateral cerebral hemispheres.

### 2.3 Image analysis

Two radiologists with extensive experience in fetal MRI diagnosis (1 associate chief physician with >15 years of work experience and 1 attending physician with >10 years of work experience) analyzed the cranial MRI images of 16 fetuses, all of whom were diagnosed with cACC combined with unilateral cortical malformation. The imaging characteristics of this specific malformation were analyzed, and the MRI findings were evaluated. Referring to a classification system from a literature in European Radiology (8), 17 categories were defined based on detailed anatomical features, among which there were 6 categories of unilateral cortical malformations: C1 (extensive cortical twisting and folding), C2 (local increase in sulci and gyri), C3 (local decrease in sulci and gyri), C4 (transcortical cleft formation), C5 (focal cortical indentation or serrated changes), and C6 (unilateral hemispheric enlargement). Among these, C1, C4, and C5 may be accompanied by corpus callosum malformation. The fetal cortical malformations in this study were classified, and the fetal biparietal diameter, gender, involved lobes, and signal characteristics, as well as other manifestations such as interhemispheric cysts, widened interhemispheric fissures, and ventricular enlargement, were summarized and analyzed. Meanwhile, the correlations with fetal gender and gestational age were explored. The final diagnostic results were obtained through consultation.

## 3 Results

### 3.1 Imaging results

#### 3.1.1 Fetal biparietal diameter and gender

The fetal biparietal diameter was within the normal range for gestational age, with 13 male fetuses (13/16) and 3 female fetuses (3/16). (The normal reference range recommended by the International Society of Ultrasound in Obstetrics and Gynecology (ISUOG).

#### 3.1.2 Direct and indirect signs of cACC

The corpus callosum was not visualized in axial, coronal, or sagittal views in all 16 cases. Among them, 15 cases were complicated with lateral ventricular dilatation, 6 cases with midline cysts, and 7 cases with widened cerebral interhemispheric fissures.

#### 3.1.3 Involved lobes and signal characteristics of cortical malformations

**Figure 1:**
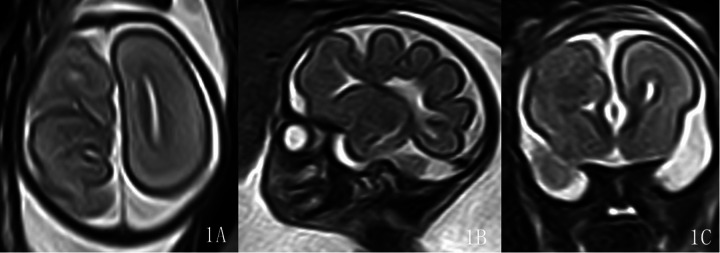
Gestational age 24 weeks, cACC, right frontoparietooccipital lobe malformation with extensive cortical twisting and folding. The ssFSE sequence shows the “rake sign” on axial view **(1A)**, the “garland sign” on sagittal view **(1B)**, and local signal reduction in the right frontal lobe with unclear gray-white matter differentiation on coronal view **(1C)**. Classification: Type I.

**Figure 2:**
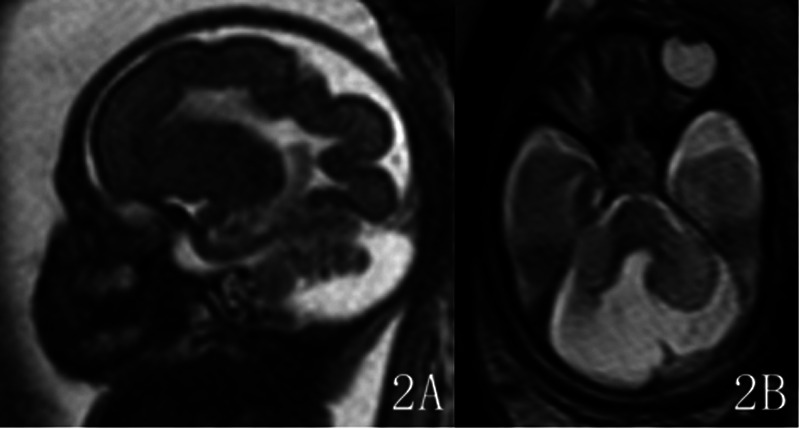
Gestational age 26 weeks, cACC. The BTFE sequence shows extensive cortical twisting and folding in the right frontoparietooccipital lobe on sagittal view, presenting the “garland sign” **(2A)**. The ssFSE sequence shows absence of the right eyeball and accompanying Dandy-Walker malformation on axial view **(2B)**. Classification: Type I.

**Figure 3:**
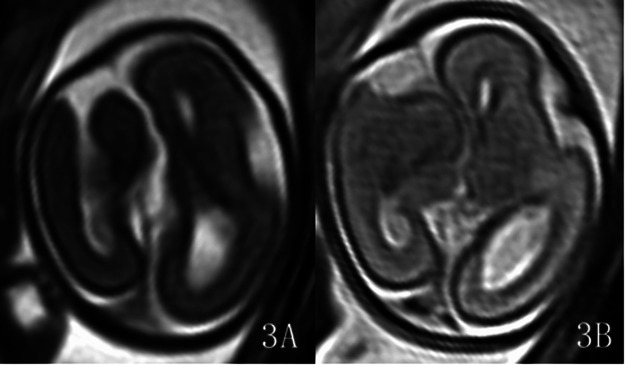
Gestational age 24 weeks, cACC. Axial view shows obvious schizencephaly in the right frontal lobe **(3A)**, and the signal of the lesioned lobe is reduced on the ssFSE sequence **(3D)**. Classification: Type II.

**Figure 4:**
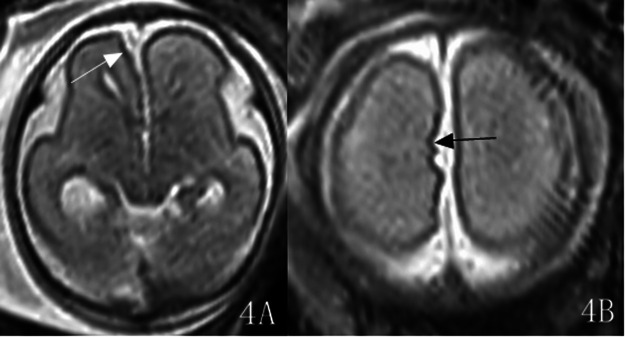
Gestational age 24 weeks and 5 days, cACC. The ssFSE sequence shows slight shrinkage of the right frontal lobe on axial view (**4A**, white arrow), and local cortical indentation in the medial part of the right frontal lobe with serrated changes (**4B**, black arrow). Classification: Type III.

The malformation was located in the left hemisphere in 7 cases and in the right hemisphere in 9 cases. The lesions involved the frontal lobe in 16 cases, among which 6 cases were limited to the frontal lobe. Ten cases involved the parietal lobe, 8 cases involved the occipital lobe, and no cases involved the temporal lobe. Classification: 11 cases of Type I (C1 type: extensive cortical twisting and folding): 11 cases showed the “rake sign” on axial views (Figure 1A), i.e., the extensively dysplastic and abnormally folded cortex on axial cranial views resembled a rake; 11 cases showed the “garland sign” on sagittal views (Figures 1B, 2A), i.e., the extensively dysplastic and abnormally folded cortex on sagittal cranial views resembled a garland. One of these cases was accompanied by Dandy-Walker malformation and absence of the right eyeball (Figure 2B). One case of Type II (C4 type: transcortical cleft formation) (Figures 3A,B). Four cases of Type III (C5 type: focal cortical indentation or serrated changes) (Figure 4), part of which were located in the medial part of the lobes, and 3 cases were accompanied by slight local shrinkage of the involved lobes (Figure 4A). In 10 cases, the signal of the involved lobes was lower than that of the contralateral cerebral parenchyma on the ssFSE sequence (Figures 1C, 2D), part of cases with unclear gray-white matter differentiation.

### 3.2 Prenatal ultrasound and amniocentesis

#### 3.2.1 Prenatal ultrasound

All cases underwent prenatal ultrasound examination before MRI. However, the ultrasound findings were summarized from the official ultrasound reports provided by the patients. Due to the limitation that high-quality ultrasound images could not be obtained, the specific ultrasound features were recorded according to the descriptions in the reports. Agenesis of the corpus callosum was diagnosed by ultrasound in all cases. Only 2/16 cases were clearly diagnosed with unilateral hemispheric cortical malformation by ultrasound, both of which were Type III.

#### 3.2.2 Amniocentesis

Three cases underwent prenatal amniocentesis, among which 1 case had a gene deletion in the chr13:73460071–73583279 segment by whole-genome chromosomal microarray analysis (CMA), and 1 case had a hemizygous variant of the NSDHL gene.

### 3.3 Prognosis

#### 3.3.1 Pregnancy choices

15/16 (93.75%) cases chose pregnancy termination after multidisciplinary prenatal consultation (radiology/ obstetrics/ neonatology/ genetics) regarding fetal malformation and potential neurodevelopmental risks; 1/16 (6.25%) chose to continue the pregnancy.

#### 3.3.2 Neonatal outcomes

The single live-born case (male, Type III malformation) is a 2-year-old child with normal language, motor, and cognitive function (comparable to age-matched peers) and no detected neurological abnormalities (e.g., epilepsy, developmental delay) to date (Table 1).

**Table 1:** Clinical and imaging characteristics of the participants with cACC in this study.

NO.	maternal age	G (weeks)	BPD (mm)	Sex	Prenatal US of Cortex	Prenatal MRI						Gene	Outcome
						Lobule of the brain	Characteristic imaging signs	Associated intracranial anomalies	ssFSE signal	Type	Other Abnor-malities		
1	27	24 ^+^ ^5^	62.8	M	-	R-FL		VD, WIF		III			TOP
2	30	25	69.1	M	+	L-FL		VD		III			TOP
3	33	24	62.1	M	+	L-FL		VD		III		+	LB
4	32	23 ^+^ ^3^	59.8	M	-	L-FL/PL	RS, GS	VD		I		+	TOP
5	31	27 ^+^ ^2^	74.1	M	-	R-FL/PL	RS, GS	VD	reduction	I			TOP
6	46	26 ^+^ ^5^	64.8	M	-	R-FL/PL	RS, GS	VD	reduction	I		-	TOP
7	34	27	70.3	M	-	R-FL/PL	RS, GS	VD, MD		I			TOP
8	29	23 ^+^ ^3^	61.2	M	-	L-FL	RS, GS	VD, MD		I			TOP
9	35	24	63.7	M	-	R-FL/PL/OL	RS, GS	VD, WIF	reduction	I			TOP
10	32	23	59.5	F	-	R-FL/PL	RS, GS	VD, MD	reduction	I			TOP
11	34	23	56.6	F	-	L-FL/PL	RS, GS	VD, WIF		I			TOP
12	27	23 ^+^ ^1^	62.3	M	+	L-PL/OL		VD, WIF		III			TOP
13	22	24 ^+^ ^5^	63.9	M	-	L-FL/PL	RS, GS	VD, MD		I			TOP
14	37	24	71.4	M	-	R-FL	RS, GS	VD, MD		I			TOP
15	26	24	59.1	F	-	R-FL		VD	reduction	II			TOP
16	27	26	64.9	M	-	R-FL/PL/OL	RS, GS	MD		I	D-WS, Right anopht-halmia		TOP

cACC, complete agenesis of the corpus callosum; MA, maternal age; F, female; M, male; GA, gestational age; BPD, biparietal diameter; TOP, termination of pregnancy; LB, Live birth; R, right; L, left; FL, frontal lobe; PL, parietal lobe; OL, occipital lobe; RS, rake sign; GS, garland sign; VD, ventricular dilatation; MD, midline cysts; WIF, widened interhemispheric fissure; D-WS, Dandy-Walk syndrome.

## 4 Discussion

### 4.1 Background and case characteristics

The corpus callosum is a white matter fiber bundle connecting the left and right cerebral hemispheres. As the most critical connection pathway, it is mainly responsible for coordinating, transmitting, and integrating information between the two cerebral hemispheres (9). Agenesis of the corpus callosum is associated with focal cortical gyral malformations and other cortical malformations, especially cACC, which has a significant correlation with cortical malformations (7). These malformations are often associated with severe neurological, cognitive, and behavioral disorders, such as epilepsy, autism, and schizophrenia (10). Previous classifications of cortical malformations mostly included lissencephaly, polymicrogyria, schizencephaly, focal cortical dysplasia, and periventricular nodular heterotopia, but the heterogeneity of this malformation is far beyond this classification (11). A study based on 356 fetal magnetic resonance cases established a classification system for fetal cortical malformations, among which one type of unilateral cortical malformation is associated with ACC (8). The main reason for selecting cACC in this study is to exclude the impact of the variability of residual segments of partial corpus callosum on the research. In addition, this study found that the gestational age of fetuses with this malformation ranged from 23 weeks to 27 weeks and 2 days. The possible reason is that ACC is usually detected after 20 weeks of gestation, and if abnormal cerebral sulci appear within this gestational age range, a diagnosis can be made. In the third trimester of pregnancy, the cerebral sulci and fissures of the normal cerebral cortex on both sides of the affected fetus are basically formed, and abnormal cerebral sulci are not easy to distinguish from them, which is prone to missed diagnosis. In the second and third trimesters of pregnancy, with the increase of gestational age, the degree of cerebral cortical folding continues to increase. The normal central sulcus, precentral sulcus, and postcentral sulcus are gradually formed after 26 weeks of gestation (12). Therefore, if abnormal cortical folding in the frontoparietal lobe is found before 26 weeks of gestation, it can indicate cortical malformation. This study found that the proportion of male fetuses with this lesion was relatively high (13/16 cases), which is consistent with the reports of previous relevant literatures (13), i.e., the genetic phenotype of ACC combined with cortical malformation is related to the X chromosome. In addition, ACC is also more common in male children, with a male proportion of 54%–73% (14).

### 4.2 Ultrasound and MRI characteristics

Prenatal ultrasound is an important screening method for fetal cerebral cortical malformations, with characteristic ultrasonic manifestations, but it is prone to missed diagnosis (15). In this study, ACC was diagnosed by prenatal ultrasound in all 16 fetuses, but the combined cortical abnormalities were only found in 2 cases. The extremely low ultrasound detection rate (2/16, 12.5%) of associated cortical malformations may be mainly due to skull acoustic shadow, fetal position limitations, and the subtlety of unilateral cortical folding abnormalities; only the relatively obvious focal cortical indentation (Type III) can be partially identified by ultrasound, while the extensive cortical twisting/folding (Type I) and transcortical cleft (Type II) are almost undetectable. The diagnostic sensitivity is lower than that of MRI. The diagnostic accuracy of prenatal brain MRI is higher than that of prenatal ultrasound (16), and MRI has higher resolution in displaying fetal cerebral cortical folding, which can more clearly show the fine structure of the cerebral cortex (17). Prenatal MRI should be routinely performed for all fetuses with ultrasound-diagnosed cACC to screen for associated cortical malformations, regardless of whether ultrasound shows other intracranial anomalies. Among the 16 lesions found in this study, Among the 16 lesions identified in this study, all involved the frontal lobe. Isolated involvement of the frontal lobe was observed in 6 cases. The parietal lobe was involved in 10 cases, while the occipital lobe was less frequently affected. The temporal lobe was not involved in any case. but there was no much difference between the left and right hemispheres, which is consistent with previous studies (13). The analysis shows that there is a significant spatial adjacency between the area of corpus callosum absence and the medial surface of the normal frontoparietal cortex. The specific mechanism of unilateral occurrence is still unclear, which may be related to the incomplete consistency of the development of the left and right cerebral cortices. Some research suggests that the fetal left and right cerebral hemispheres do not exhibit absolute structural and functional homology during mid-trimester cortical formation (20–28 weeks of gestation), the critical window for sulcal and gyral development. This intrinsic developmental asynchrony is a physiological characteristic of fetal brain development (18). And the absence of the corpus callosum (the primary structural and molecular connection between the two hemispheres) disrupts the bidirectional crosstalk of developmental signals (e.g., growth factors, transcription factors) between the hemispheres. Unilateral hemispheric cortical impairment of processes easily leads to focal cortical malformation due to the loss of corpus callosum-mediated regulatory signals (3, 19). In addition, partial ACC-associated midline anomalies (e.g., midline cysts, widened interhemispheric fissures) in our cases may cause unilateral intracranial hemodynamic changes and spatial compression, further aggravating cortical developmental abnormalities on the affected side. As for whether the unilaterally abnormally folded cortex can develop and improve again in the third trimester of pregnancy, some studies have found that some cases were examined in the third trimester and only showed unilateral abnormalities. It is believed that the development of the unilaterally abnormally folded cortex is synchronized with the development of the corpus callosum in time and will not change in the third trimester (20).

According to the existing classification standards and characteristics, all cases can be divided into three types. Among them, Type I (C1 type) is the most common, manifested as extensive cortical twisting and folding. We also named them the “rake sign” and “garland sign” according to the special morphological characteristics of this type on axial and sagittal cranial views, which are characteristic on MRI. Type II is relatively rare, accompanied by transcortical cerebral cleft formation. Type III is characterized by focal cortical indentation, with local sulci and gyri showing serrated changes, mostly located in the medial part of the lobes. This study found that the signal of some lesions on the ssFSE sequence was lower than that of the contralateral normal cerebral parenchyma, with unclear gray-white matter differentiation, which may be related to abnormal neuronal migration. In addition, it is often accompanied by other manifestations, such as midline cysts, widened interhemispheric fissures, and lateral ventricular dilatation, which are also common accompanying signs of ACC.

### 4.3 Prognosis and amniocentesis

In this study, 15 fetuses were aborted by their parents after MRI diagnosis, and only 1 case of Type III was live-born, now 2 years old. This may be because the cortical abnormal folding shown by this type on MRI is milder than that of the other two types, so the prognosis is acceptable. Therefore, it is suggested that children with Type III may have a relatively better clinical prognosis. Studies have shown that about 2/3 of children with cACC have a good developmental outcome, but these children may have varying degrees of defects, which are more obvious at 10–20 years old, including neuropsychological disorders and behavioral disorders (21). CACC associated with cortical dysplasia has been found to have a worse neurodevelopmental prognosis than isolated ACC (10). However, whether unilateral cortical malformation will lead to a worse prognosis of fetuses with complete ACC, or whether the results of such cases may overlap with those of isolated cACC cases, requires further large-scale prospective studies to evaluate the neurodevelopmental outcomes of fetuses (22). Only 3 cases in this study underwent amniocentesis, 1 case had a hemizygous variant of the NSDHL gene. Some research suggests that NSDHL gene can encode NAD(P)H steroid dehydrogenase-like protein, a key enzyme in the cholesterol biosynthesis pathway. Cholesterol is a critical component of neuronal cell membranes and myelin sheaths; dysregulation of the NSDHL gene leads to abnormal cholesterol synthesis, which impairs neuronal migration, cortical folding, and white matter fiber bundle formation during fetal brain development (23–25). The hemizygous NSDHL variant in the study may contribute to both cACC (abnormal white matter fiber formation) and unilateral cortical malformation (impaired regional neuronal migration/cortical development), consistent with the gene's established role in CNS development. Imaging examinations can diagnose functional abnormalities of fetal cortical malformations. Clinical diagnosis combined with genetic testing is a method that can achieve early diagnosis and evaluate whether fetuses have functional abnormalities, and is one of the important means of prenatal diagnosis (26).

## 5 Conclusions

The most important clinical finding of this study is that prenatal ultrasound has a remarkably low detection rate for cACC-associated unilateral hemispheric cortical malformations, and thus routine prenatal MRI is indicated for all fetuses with ultrasound-diagnosed cACC to screen for associated cortical malformations and optimize perinatal management. In addition, prenatal MRI can accurately diagnose fetal ACC combined with unilateral cortical malformation, which has the following characteristics: ① It is usually detected around 25 weeks of gestation; ② It is relatively more common in boys; ③ It most often involves the frontal lobe, among which the “rake sign” and “garland sign” have certain auxiliary diagnostic significance. ACC combined with unilateral cortical malformation has great uncertainty in long-term prognosis, and most prospective parents will consider terminating the pregnancy. Prenatal MRI can accurately identify the presence, side, location and subtype of associated unilateral cortical malformations, which is critical for the multidisciplinary team (radiologists, obstetricians, geneticists and neonatologists) to conduct comprehensive prognosis assessment, provide detailed parental counseling, and formulate individualized perinatal management plans.

## 6 Limitations

This study has several limitations. First, it adopted a single-center retrospective design, which may restrict data comprehensiveness and introduce inherent retrospective biases. Results have limited generalizability to other populations, as the sample was from a single center and may not represent broader groups with similar conditions. Second, the relatively small sample size may reduce statistical power and affect result reliability. Third, the lack of long-term postnatal neurodevelopmental follow-up prevented evaluation of long-term lesion outcomes and impacts on children's growth. Additionally, we did not collect detailed information on maternal confounding factors (e.g., maternal age, gestational diabetes, and medication use during pregnancy), which may have potential impacts on the research results. Finally, the diagnostic criteria for partial lesions were based on imaging findings alone, without pathological confirmation, which may lead to minor deviations in diagnostic accuracy.

## 7 Future work

This study systematically characterizes the prenatal MRI features of fetal complete agenesis of the corpus callosum (cACC) combined with unilateral hemispheric cortical malformation, but several important research gaps remain to be addressed. Future studies will expand the sample size, adopt a multi-center design to improve data representativeness, and conduct long-term postnatal follow-up of live-born children to clarify prognosis and long-term neurodevelopmental outcomes. We will also collect comprehensive maternal confounding factors and combine pathological examinations to improve diagnostic accuracy, thereby enhancing the research's scientificity and applicability. Advanced neuroimaging (e.g., DTI, fMRI) will investigate brain connectivity/functional networks in live-born children, informing optimized rehabilitation strategies.

## Data Availability

The original contributions presented in the study are included in the article/Supplementary Material, further inquiries can be directed to the corresponding author.
