# Supplementary figures and images for: Prenatal MRI features of fetal complete agenesis of the corpus callosum associated with unilateral hemispheric cortical malformation: a retrospective study
Source: Front Radiol. 2026 May 1;6:1802335. doi: 10.3389/fradi.2026.1802335 (PMC13175969; doi:10.3389/fradi.2026.1802335)

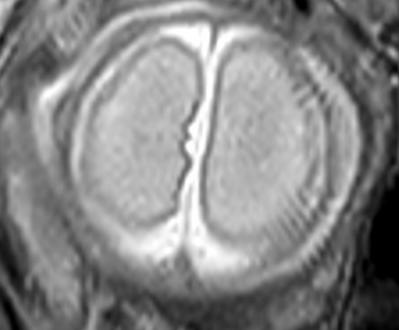

Supplement: Supplementary file 1 [file Datasheet1.zip › MRI figures of all cases/Case 1.jpg]

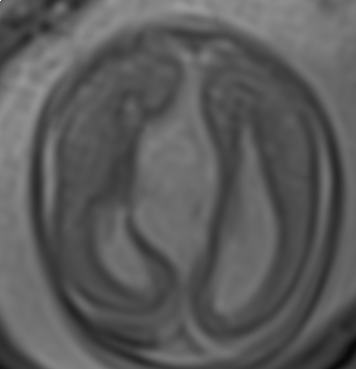

Supplement: Supplementary file 1 [file Datasheet1.zip › MRI figures of all cases/Case 10.jpg]

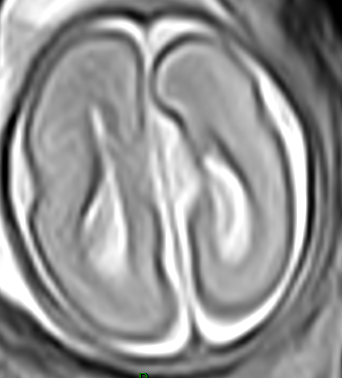

Supplement: Supplementary file 1 [file Datasheet1.zip › MRI figures of all cases/Case 11.jpg]

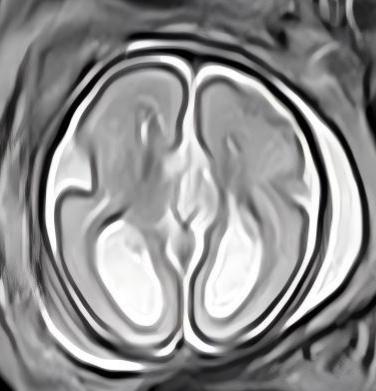

Supplement: Supplementary file 1 [file Datasheet1.zip › MRI figures of all cases/Case 12.jpg]

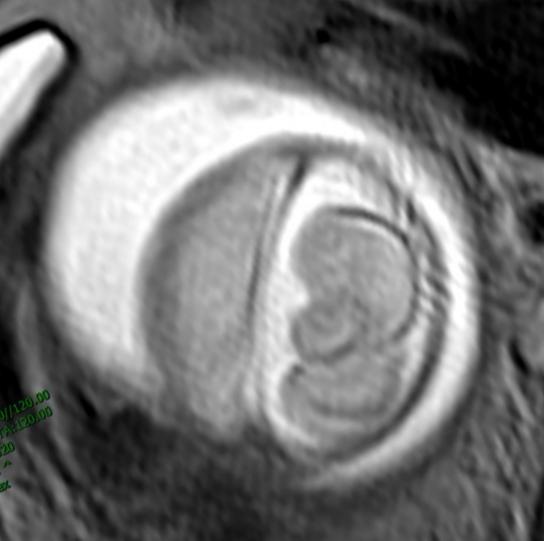

Supplement: Supplementary file 1 [file Datasheet1.zip › MRI figures of all cases/Case 13.jpg]

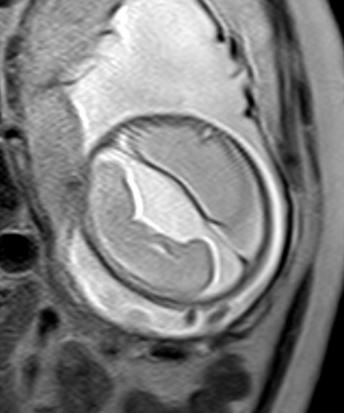

Supplement: Supplementary file 1 [file Datasheet1.zip › MRI figures of all cases/Case 14.jpg]

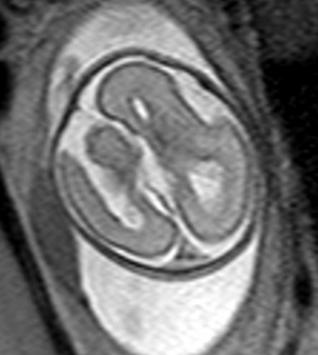

Supplement: Supplementary file 1 [file Datasheet1.zip › MRI figures of all cases/Case 15.jpg]

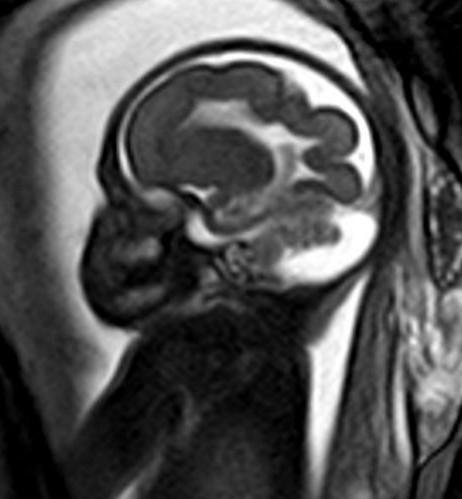

Supplement: Supplementary file 1 [file Datasheet1.zip › MRI figures of all cases/Case 16.jpg]

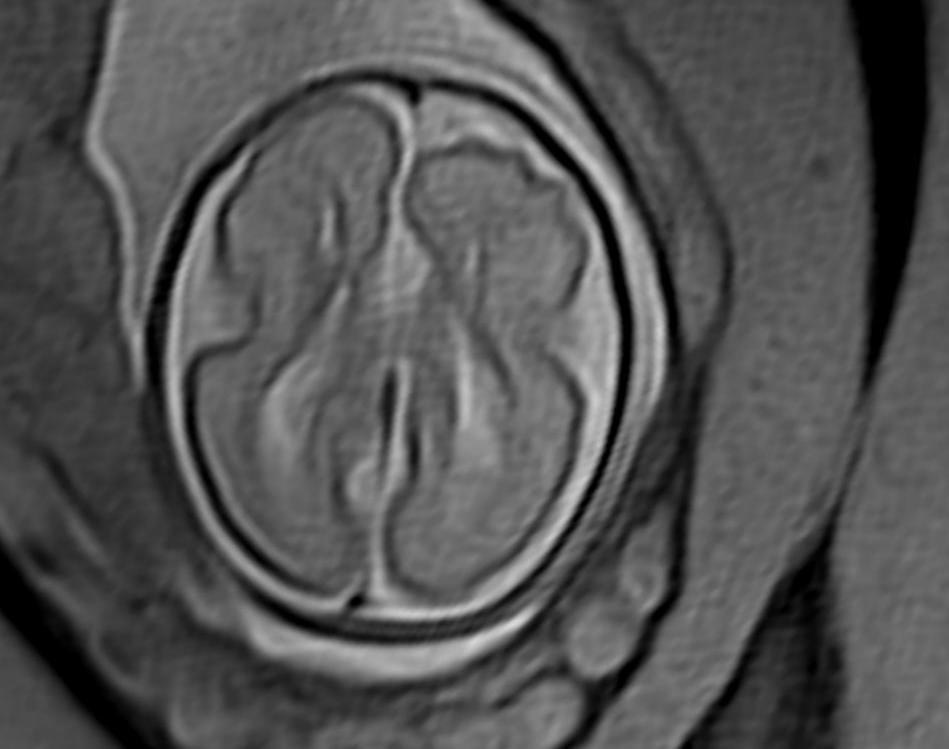

Supplement: Supplementary file 1 [file Datasheet1.zip › MRI figures of all cases/Case 2.jpg]

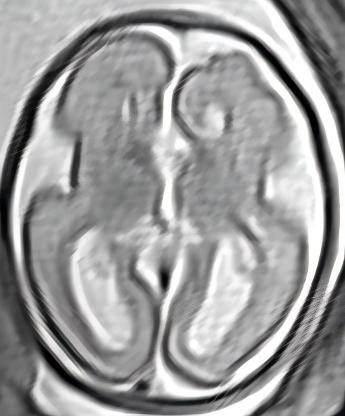

Supplement: Supplementary file 1 [file Datasheet1.zip › MRI figures of all cases/Case 3.jpg]

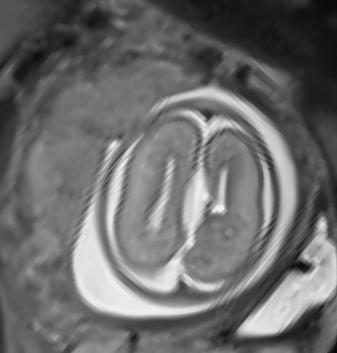

Supplement: Supplementary file 1 [file Datasheet1.zip › MRI figures of all cases/Case 4.jpg]

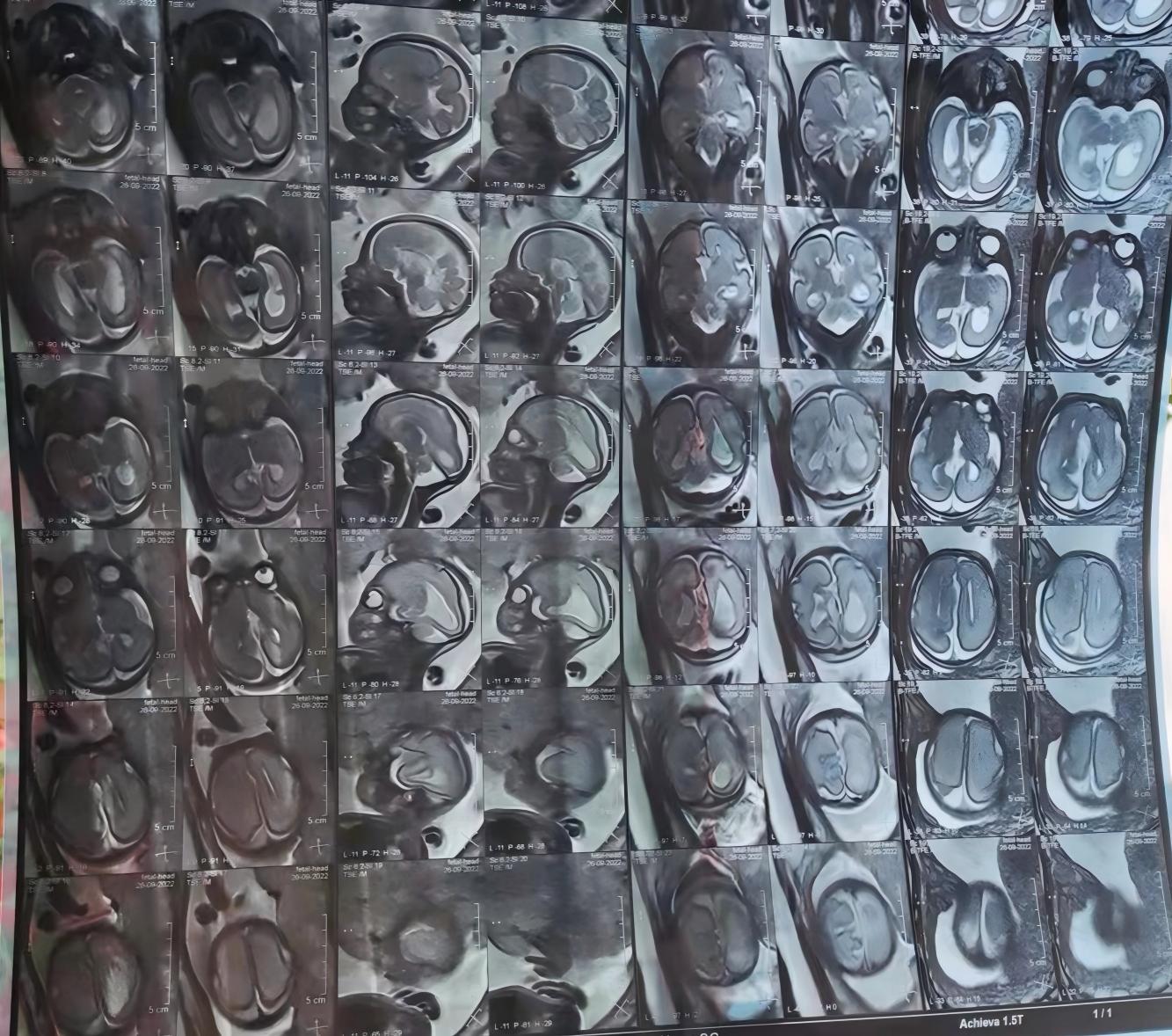

Supplement: Supplementary file 1 [file Datasheet1.zip › MRI figures of all cases/Case 5.jpg]

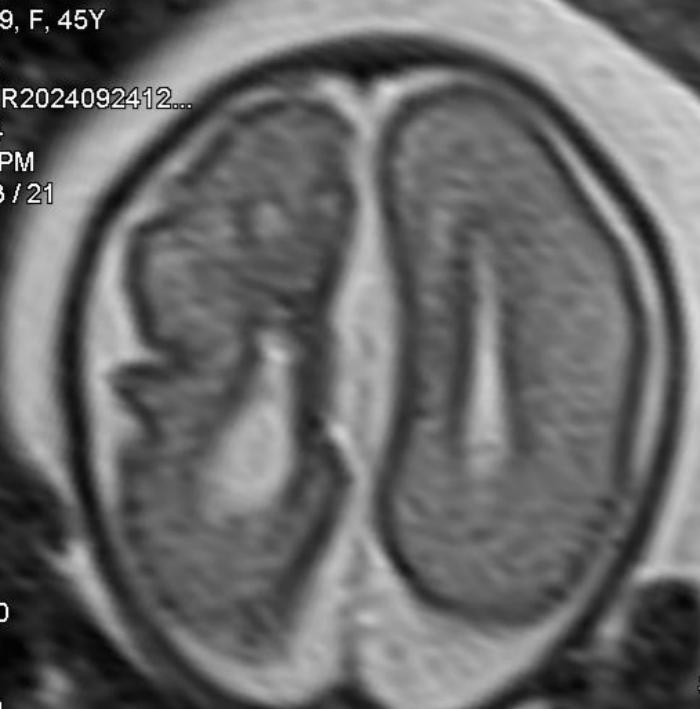

Supplement: Supplementary file 1 [file Datasheet1.zip › MRI figures of all cases/Case 6.jpg]

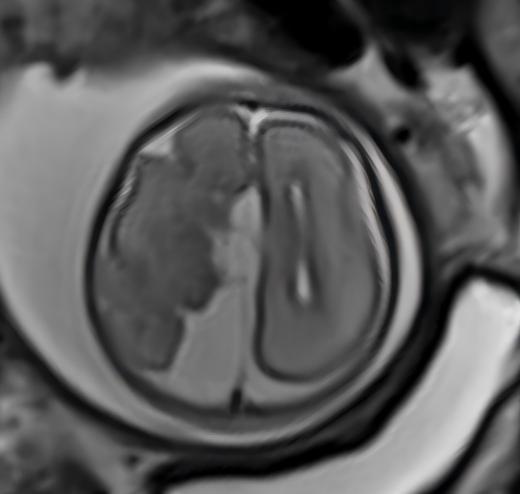

Supplement: Supplementary file 1 [file Datasheet1.zip › MRI figures of all cases/Case 7.jpg]

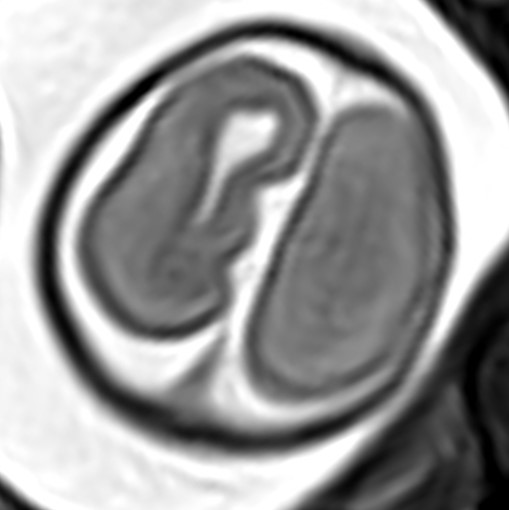

Supplement: Supplementary file 1 [file Datasheet1.zip › MRI figures of all cases/Case 8.jpg]

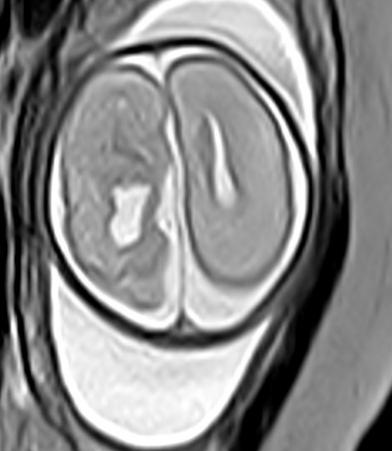

Supplement: Supplementary file 1 [file Datasheet1.zip › MRI figures of all cases/Case 9.jpg]
